# Neurofeedback: Applications, advancements, and future directions

**DOI:** 10.1002/pcn5.70259

**Published:** 2025-12-25

**Authors:** Hassan Jubair, Mithela Mehenaz, Md. Merajul Islam, Nilufa Yeasmin

**Affiliations:** ^1^ Department of Electrical and Electronic Engineering Khulna University of Engineering and Technology Khulna Bangladesh; ^2^ Department of Electrical and Electronic Engineering Varendra University Rajshahi Bangladesh; ^3^ Department of Electrical and Electronic Engineering Rajshahi University of Engineering and Technology Rajshahi Bangladesh; ^4^ Department of Obstetrics & Gynecology, Rajshahi Medical College Hospital Rajshahi Bangladesh

**Keywords:** biofeedback, neurofeedback, neurofeedback techniques

## Abstract

Neurofeedback, a technique enabling individuals to regulate their brain activity in real time, has gained momentum as both a clinical intervention and a tool for cognitive and performance enhancement. This review synthesizes findings from 65 studies to evaluate the current state of neurofeedback research. We outline its historical development, methodological approaches, and technological innovations, including advances in connectivity‐based and multimodal feedback paradigms. Applications across clinical disorders, such as attention‐deficit/hyperactivity disorder (ADHD), post‐traumatic stress disorder (PTSD), depression, and autism, as well as performance optimization, are critically examined, with emphasis on efficacy, limitations, and translational challenges. To enhance transparency, we summarize methodological trends and provide integrative insights that cut across individual studies. We further discuss persistent limitations, including methodological heterogeneity and placebo‐related concerns, and highlight future directions such as personalization, multimodal integration, and interdisciplinary collaboration. By consolidating evidence across diverse domains, this review positions neurofeedback as a rapidly evolving field with significant therapeutic and translational potential.

## INTRODUCTION

Neurofeedback, a form of biofeedback that trains individuals to modulate their brain activity through real‐time feedback, has become a focal point of both clinical and cognitive research. Its appeal lies in its non‐invasive nature and capacity to directly target neural circuits implicated in psychiatric and neurological conditions, as well as in cognitive enhancement and performance optimization.^1^

Technological advances have transformed neurofeedback from early electroencephalography (EEG)‐based protocols into more sophisticated paradigms employing functional magnetic resonance imaging (fMRI) and functional near‐infrared spectroscopy (fNIRS).^2^, ^3^ Innovations such as connectivity‐based feedback^4^ and multivoxel decoding approaches^5^ now allow modulation of distributed brain networks rather than isolated regions. Moreover, the integration of machine learning and immersive platforms such as virtual reality (VR) has opened new avenues for individualized and ecologically valid interventions.^6^ A detailed account of the historical trajectory of neurofeedback is provided in the Historical Background of Neurofeedback section.

Despite these advances, methodological challenges persist. Small sample sizes, heterogeneous protocols, and limited replication reduce generalizability.^7^ Placebo effects and non‐specific factors further complicate the interpretation of efficacy.^8^, ^9^ Addressing these barriers is essential for establishing neurofeedback as a reliable therapeutic and performance‐enhancing tool.

This review focuses primarily on four conditions: attention‐deficit/hyperactivity disorder (ADHD), post‐traumatic stress disorder (PTSD), depression, and autism spectrum disorder (ASD). These disorders were prioritized due to their high prevalence, profound impact on quality of life, and strong neurobiological underpinnings that make them suitable targets for neurofeedback. For instance, ADHD affects approximately 5%–7% of children worldwide,^10^ with core deficits linked to aberrant oscillatory dynamics amendable to EEG‐based feedback.^11^ PTSD, with a lifetime prevalence near 8%,^12^ is characterized by dysregulated fear circuitry that fMRI‐based feedback seeks to normalize.^13^ Depression and ASD likewise represent major global health burdens, with disrupted prefrontal–limbic regulation and atypical connectivity patterns, respectively, forming mechanistic rationales for neurofeedback intervention.^14^ Anchoring this review in these domains ensures clinical relevance while facilitating synthesis across the most extensively researched applications.

## METHODS

### Inclusion and exclusion criteria

Studies were included if they met the following criteria: (1) examined the application or advancement of neurofeedback techniques, (2) were published in English, (3) involved human participants, (4) presented empirical data on neurofeedback efficacy or outcomes, (5) provided sufficient methodological detail to assess study quality, and (6) reported quantifiable results (e.g., accuracy, effectiveness, or clinical/behavioral outcomes). Studies identified through reference list cross‐checking were subjected to the same criteria.

Exclusion criteria were the following: (1) single‐subject case reports, (2) studies involving participants with comorbid conditions (e.g., chronic cardiovascular, renal, or metabolic disease) that could confound outcomes, and (3) conference abstracts or proceedings unless an extended peer‐reviewed journal version was available.

### Search strategy

Given the interdisciplinary scope of neurofeedback research, five electronic databases were searched: PubMed, Scopus, IEEE Xplore, Web of Science, and PsycINFO. The search covered studies published between January 2000 and March 2024, restricted to English‐language publications involving human participants.

#### PubMed search string

The following Boolean expression was applied, with MeSH terms when available:

(“Neurofeedback” [MeSH Terms] OR neurofeedback OR “EEG neurofeedback” OR “fMRI neurofeedback”

OR “real‐time fMRI” OR “functional connectivity neurofeedback” OR “brain‐computer interface”

OR “BCI” OR “biofeedback” OR “neurotherapy” OR “brain training”)

AND

(“clinical application” OR “cognitive enhancement” OR “psychiatric disorders”

OR “ADHD” OR “PTSD” OR “depression” OR “anxiety” OR “autism” OR “performance optimization”

OR “sports training”)

AND

(“humans” [MeSH Terms])

AND

(“2000/01/01” [Date ‐ Publication]: “2024/03/31” [Date ‐ Publication])

AND

(English [lang])

Equivalent keyword structures were adapted for other databases, with Boolean operators (AND/OR) and truncation symbols (e.g., “neurofeed*”) used to maximize retrieval sensitivity.

All references were exported into EndNote for deduplication. Titles and abstracts were screened first, followed by full‐text reviews against inclusion/exclusion criteria. Reference lists of included studies were manually searched to identify additional eligible papers.

### Extraction of study characteristics

For each included study, the following information was extracted: author(s) and year of publication, population characteristics, sample size, neurofeedback modality (e.g., EEG, fMRI, and fNIRS), study design, primary outcomes, and key methodological details (e.g., protocols, control conditions). Reported quantitative measures (e.g., effect sizes, accuracy, and clinical improvement scores) were recorded and summarized in tabular format.

### Preferred Reporting Items for Systematic reviews and Meta‐Analyses flow and study selection

**Figure 1:**
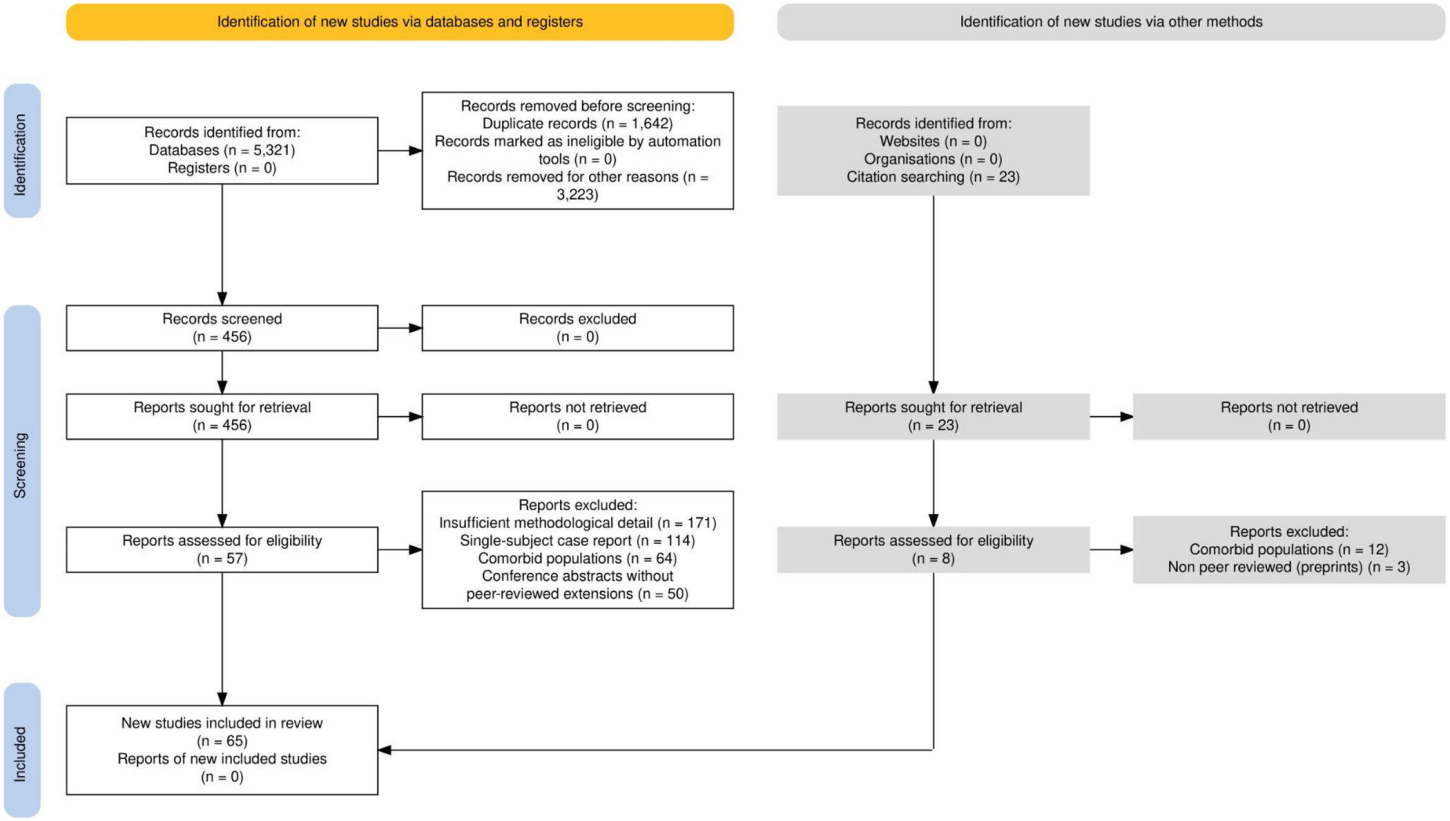
Flow diagram of the systematic review process.

The study selection process is summarized in Figure 1 (Preferred Reporting Items for Systematic reviews and Meta‐Analyses [PRISMA] flow diagram). A total of 5321 records were retrieved. After title and abstract screening, 4865 studies were excluded for irrelevance (3223) and duplication (1642). The remaining 456 full‐text articles were assessed for eligibility. Manual cross‐referencing of bibliographies yielded an additional 23 studies, for a total of 479 full‐texts reviewed. The final synthesis included 65 studies.

The systematic review process is summarized in the PRISMA flow diagram in Figure 1.

## RESULTS

### Historical background of neurofeedback

Neurofeedback, also known as EEG biofeedback or neurotherapy, traces its roots back to the pioneering work of researchers in the mid‐20th century. The concept of neurofeedback emerged from studies investigating the brain's electrical activity and its potential modulation through operant conditioning.^15^

One of the earliest proponents of neurofeedback was Joe Kamiya, whose experiments in the 1960s laid the foundation for the field. Kamiya demonstrated that individuals could learn to control their brainwave patterns, particularly alpha waves, through feedback mechanisms.^16^ His research sparked interest in the possibility of using neurofeedback for therapeutic purposes.

Building upon Kamiya's work, Barry Sterman conducted groundbreaking studies in the 1970s, focusing on the application of neurofeedback in epilepsy management.^16^ Sterman discovered that cats trained to increase sensorimotor rhythm (SMR) brainwaves exhibited reduced susceptibility to seizures.^17^ This discovery paved the way for the development of neurofeedback protocols for epilepsy patients, offering a non‐pharmacological approach to seizure control.

During the same period, researchers such as Joel Lubar and John F. Lubar explored neurofeedback's potential in addressing ADHD.^18^ Their studies demonstrated that children with ADHD could learn to regulate their brain activity and improve attention and impulse control through neurofeedback training.^19^

The 1980s witnessed further advancements in neurofeedback technology, with the introduction of computerized EEG systems and sophisticated feedback displays. These technological innovations facilitated more precise and real‐time monitoring of brain activity, enhancing the efficacy and accessibility of neurofeedback interventions.^6^, ^20^, ^21^

As neurofeedback gained recognition as a viable therapeutic modality, researchers began exploring its applications across a wide range of neurological and psychiatric conditions.^17^, ^21^ Studies in the 1990s and 2000s investigated the efficacy of neurofeedback in treating conditions such as anxiety disorders, depression, PTSD, and ASD.^22^, ^23^, ^24^, ^25^, ^26^, ^27^, ^28^, ^29^, ^30^, ^31^

In recent years, neurofeedback has undergone significant refinement and diversification, with the advent of advanced imaging techniques such as fMRI and fNIRS. These neuroimaging modalities offer insights into brain function at a higher spatial resolution, enabling researchers to target specific brain regions and networks with greater precision.^32^

Overall, the historical trajectory of neurofeedback reflects a remarkable evolution from its humble beginnings as an experimental technique to its current status as a clinically validated therapeutic approach.^29^ By understanding the historical context of neurofeedback development, researchers can appreciate the complexities of brain–behavior interactions and harness the full potential of this transformative technology. Table 1 shows a summary of included neurofeedback studies.

**Table 1:** Summary of included neurofeedback studies: populations, modalities, designs, and outcomes.

Authors^reference^	Year	Population	Neurofeedback modality	Design	Outcomes
Watanabe et al.^4^	2017	Mixed (review of healthy and clinical)	fMRI (DecNef, FCNef)	Review	Advanced understanding of implicit NF, causal brain–behavior links, but mechanisms unclear
	2015	Attention‐deficit/hyperactivity disorder (ADHD) (children, adolescents)	EEG‐NF, fMRI‐NF	Review of clinical studies	Promising non‐pharma treatment for ADHD, but studies lack rigor and consistency
Kaur et al.^3^	2019	General (clinical + healthy)	EEG‐NF (LORETA, live *z*‐scores)	Review	Enhances cognition, treats disorders, needs better targeting and controlled studies
Shibata et al.^33^	2019	Healthy	Decoded fMRI‐NF	Empirical + modeling	DecNef triggers targeted brain activity and behavior changes via implicit learning
Pandria et al.^7^	2020	Smokers	EEG‐NF, fMRI‐NF, BF	Systematic review	NF/BF can modulate cravings and CNS activity; individualized NF may improve outcomes
Lau‐Zhu et al.^34^	2019	PTSD (*n* = 4 in case study)	Decoded fMRI‐NF	Systematic review + case study	DecNef reduced PTSD severity; avoids conscious exposure but limited data
Paret et al.^6^	2019	Healthy and clinical populations (varied age groups)	Real‐time fMRI‐based neurofeedback (rtfMRI‐NF)	Narrative/methodological review	Feasible across populations; region‐specific modulation possible; personalization and training improvements noted
Walker^35^	2009	Mixed neurological and psychological disorders	Quantitative electroencephalography (QEEG)‐guided neurofeedback	Observational, clinical experience	Effective personalization for dysfunction types (hypo‐, hyper‐, disconnections); improved treatment targeting
Thibault and Raz^1^	2017	General overview	General EEG‐based neurofeedback	Literature review	Emphasizes lack of experimental rigor; questions clinical efficacy; calls for sham controls
Masterpasqua and Healey^36^	2003	Primarily ADHD	EEG neurofeedback (theta/beta training)	Review of clinical trials	Effective for ADHD; encourages psychologist use; less evidence for other disorders
Thatcher et al.^37^	2020	Clinical (e.g., ADHD, TBI)	EEG (*z*‐score, LORETA)	Technical review	Targets brain hubs, improves specificity, real‐time feedback
Ioannides^38^	2018	General/clinical	fMRI‐NF	Theoretical	Modulates large networks, restores self‐representation
Tachibana^39^	2018	Theoretical/societal	fMRI‐NF	Ethical analysis	NF blurs mental/social/moral lines; calls for ethical oversight
Jeunet et al.^40^	2018	Clinical + BCI users	EEG‐NF + BCI	Literature review	Cognitive/motivational/tech factors affect NF; BCI can inform NF
Thibault et al.^9^	2018	EEG‐NF researchers	EEG‐NF	Critical review	Research lacks rigor; influenced by ideology and commerce
Sherlin et al.^41^	2011	Clinical	EEG‐NF	Theoretical	NF must follow operant learning principles; many studies fall short
Thibault et al.^17^	2015	General	EEG	Theoretical review	NF shows promise; evidence unclear; needs rigorous studies
Hammond^23^	2005	Anxiety, PTSD, OCD, depression	EEG	Narrative review	Improvements in brain function; OCD outcomes mixed
Gruzelier^42^	2005	General/clinical	EEG	Review of validation studies	Shows efficacy; emphasizes experimental rigor
Watanabe et al.^4^	2017	General/clinical	fMRI (DecNef, FCNef)	Narrative review	Shows cutting‐edge advances in causal neural training
	2015	ADHD	EEG, fMRI	Literature review	NF is promising for ADHD; needs more rigorous studies
Kaur et al.^3^	2019	General	EEG‐NF (LORETA, *z*‐score)	Review	NF shows promise; needs better spatial resolution and larger studies
Shibata et al.^33^	2019	General	DecNef (fMRI)	Theoretical + experimental	Induces neural activity unconsciously; behavioral effects noted
Pandria et al.^7^	2020	Smokers	EEG‐NF, BF, fMRI‐NF	Review	NF/BF helps modulate cravings; influenced by individual differences
Lau‐Zhu et al.^34^	2019	PTSD (4 patients)	DecNef (fMRI)	Systematic review + pilot study	PTSD symptoms reduced; exposure not needed; promising but early
Paret et al.^6^	2019	Broad (clinical + non‐clinical)	fMRI‐NF	Review	fMRI‐NF effective; lacks standard metrics and guidelines
Walker^35^	2009	Neuro/psych disorders	QEEG‐guided EEG‐NF	Review	QEEG improves targeting; custom NF protocols needed
Thibault et al.^20^	2016	General	Mixed (EEG/fMRI)	Critical review	NF promising but lacks strong empirical basis
Masterpasqua and Healey^36^	2003	ADHD	EEG‐NF	Review	Effective for ADHD; early stage for others
Thatcher et al.^37^	2020	Various	LORETA EEG‐NF (19 ch)	Technical review	Targeting brain hubs enhances efficacy
*Frontiers in Human Neuroscience*	2018	General	fMRI/EEG‐NF (implied)	Conceptual paper	NF may restore self‐representation; ethical concerns
Tachibana^39^	2018	General	fMRI‐NF	Ethical analysis	Raises questions on enhancement and moral implications
Jeunet et al.^40^	2018	General clinical	EEG‐NF, informed by BCI	Short review	Task/motivation/tech factors from BCI can improve NF efficacy
Thibault et al.^9^	2018	NF research community	EEG‐NF	Commentary	Criticizes lack of rigor, commercial bias, and poor controls
Sherlin et al.^41^	2011	Clinical populations	EEG‐NF	Theoretical review	NF must align with learning theory; current designs often deviate
Thibault et al.^17^	2015	General	EEG‐NF	Review	NF potential overstated; calls for better design and follow‐up
Hammond^23^	2005	Anxiety, PTSD, OCD, depression	EEG‐NF	Narrative review	Promising results; needs RCTs and larger studies
Gruzelier^42^	2005	Music students, healthy controls	EEG—alpha‐theta, SMR/beta	Validation review	Operant alpha‐theta control; performance gains in music
Jiang et al.^43^	2017	Older adults	EEG‐based NF	Review	NF can enhance attention and working memory. Combining traditional and NF‐based cognitive training shows promise
Flanagan and Saikia^44^	2023	General population (consumer use)	EEG and fNIRS (consumer‐grade)	Review	Highlights potential of consumer NF for mental health. Addresses challenges in accuracy, data quality, standardization, and usability
Linden^24^	2014	Individuals with depression	EEG and fMRI	Review	Advances in imaging/NF are promising for treating affective disorders. Early fMRI‐NF trials show clinical benefits
Linden et al.^45^	2012	Depressed patients	fMRI‐based NF	Experimental (non‐blinded control)	Patients learned to upregulate emotion‐related regions, improving depression symptoms (HDRS scores)
Castrén^31^	2013	Adults with depression	Not NF (focus on antidepressant + plasticity)	Conceptual review	Antidepressants enhance plasticity; combined with rehab, this may promote network recovery. Emphasizes neuroplasticity's role in recovery
Hamilton et al.^25^	2016	Female MDD patients	fMRI‐based NF (targeting Salience Network)	Experimental (real vs. sham NF)	Real NF led to reduced SN activation and lower negative emotional response. Supports SN's role in affective bias
Tucker et al.^26^	2003	Depressed versus controls	EEG (ERP analysis, not NF per se)	Lab study using video game task	Moderately depressed individuals showed exaggerated medial frontal response to negative feedback; suggests sensitized limbic network
Li et al.^27^	2018	Individuals with depression	Not NF‐specific; focus on brain networks	Review/theoretical model	Identifies 4 key brain networks involved in depression. Antidepressants can restore network connectivity. Emphasizes functional dysconnectivity
Yadollahpour and Arani^28^	2015	Depressed patients	EEG‐NF	Review	EEG‐NF is a non‐invasive, drug‐free treatment aiming to shift brain from disordered to normalized state. Protocols focus on brainwave regulation
	2013	General/clinical	EEG‐NF	Conceptual/review	Neurofeedback helps integrate first‐person experience with neural data. Promotes self‐awareness and self‐regulation. Shows promise for therapy
Thibault et al.^17^	2015	Various mental disorders	EEG‐NF, fMRI‐NF, and fNIRS	Review/critical analysis	EEG‐NF lacks strong clinical evidence; fMRI‐NF shows promise, but data are limited. Need for rigorous trials and better controls
Thibault and Raz^1^	2017	General (clinical applications)	EEG‐NF, fMRI‐NF	Review	Discusses historical and clinical context. Points out lack of methodological rigor and insufficient evidence for clinical efficacy. Calls for better design
Thompson et al.^16^	2023	Individuals with ADHD	QEEG‐based neurofeedback	Review	Explains how to customize a neurofeedback intervention to improve brain function in a harm‐free and lasting way
Coben et al.^29^	2009	Individuals with autism spectrum disorder (ASD)	EEG‐NF	Literature review	NF may enhance neuroregulation and metabolic function in ASD. Shows promise, but literature is limited. Recommends further research
Tolin et al.^30^	2019	Individuals with anxiety disorders	Biofeedback and neurofeedback	Systematic review (quant. and qual.)	Biofeedback outperforms waitlist, but not other active treatments. Difficult to separate specific effects from placebo. Overall study quality is weak
Mirifar et al.^46^	2017	Athletes	EEG‐NF	Systematic review	NF shows potential to enhance brain oscillation regulation and athletic performance; guidelines for future research proposed
Sharon^47^	2013	Individuals with various neurological/psychiatric disorders	EEG‐NF	Review	NF shows promising efficacy; mechanisms include neuroplasticity, connectivity changes, and network modulation
Linden et al.^45^	2016	Patients with Parkinson's disease and stroke	fMRI‐NF	Review	rtfMRI‐NF can promote self‐regulation and behavioral improvement in motor rehabilitation. Potential for neuroplasticity noted
Ros et al.^48^	2020	General/clinical NF researchers	All NF modalities	Consensus checklist	Developed CRED‐nf checklist for consistent NF reporting and design; emphasized need for rigorous methodology to distinguish true NF effects.
Coben et al.^29^	2009	Individuals with ASD	EEG‐NF	Literature review	NF may improve neuroregulation and metabolic function in ASD. Shows therapeutic promise; more research needed
Omejc et al.^49^	2018	Clinical and healthy populations	EEG‐NF	Review	EEG‐NF helps with self‐regulation and has cognitive/therapeutic benefits. Studies limited by design variability and lack of standardized protocols
Larsen et al.^50^	2013	Individuals with CNS dysregulation	EEG‐NF	Narrative review	NF beneficial for CNS disorders, especially in treatment‐resistant cases. Clinical support exists, but controlled studies are scarce
Fovet et al.^51^	2017	No specific population (commentary article)	General discussion, mainly EEG‐based neurofeedback	Commentary/opinion (not an empirical study)	Argues that double‐blind designs should not replace investigation of neurophysiological mechanisms; calls for a balanced approach combining clinical trial rigor with mechanistic research
Kim et al.^52^	2015	14 nicotine‐dependent smokers	rtfMRI, using activity‐only versus activity + functional connectivity (FC) feedback	Randomized between‐group design, 2 sessions, pre‐/post‐craving ratings	FC‐based feedback led to greater brain modulation and significantly larger reductions in cigarette craving than activity‐only feedback
Mehran et al.^53^	2014	24 healthy high‐IQ young adults	EEG neurofeedback (theta/beta training) with or without local sinusoidal extremely low frequency magnetic field (LSELF‐MF) exposure	Randomized, 10 sessions, experimental (NF + LSELF‐MF) versus sham (NF only)	Both groups improved, but the experimental group showed significantly greater reductions in theta/beta ratio—indicating enhanced attention and improved NF efficacy with LSELF‐MF
Alkoby et al.^54^	2018	General‐various individuals from EEG neurofeedback studies	EEG neurofeedback	Literature review on neurofeedback inefficacy and predictors of success	Identifies high variability in response; suggests psychological and neurophysiological factors predict who benefits; recommends individualized protocols to improve effectiveness

Abbreviations: BCI, brain–computer interface; CRED‐nf checklist, Consensus on the reporting and experimental design of clinical and cognitive‐behavioural neurofeedback studies; EEG, electroencephalography; fMRI, functional magnetic resonance imaging; MDD, major depressive disorder; NF, neurofeedback; PTSD, post‐traumatic stress disorder; SMR, sensorimotor rhythm.

### Methods and techniques in neurofeedback

Neurofeedback, a burgeoning field at the intersection of neuroscience and technology, employs various methodologies and techniques to modulate brain activity and promote self‐regulation. Drawing insights from the summary and key findings of 65 papers spanning diverse neurofeedback applications, we delve into the methods and techniques commonly employed in neurofeedback research and clinical practice.

#### EEG neurofeedback

EEG neurofeedback stands as a cornerstone in the field, leveraging real‐time feedback of electrical brain activity recorded from the scalp. Across the reviewed papers, EEG neurofeedback emerged prominently, particularly in studies targeting conditions such as ADHD, PTSD, insomnia, and depression.^3^, ^32^, ^38^, ^55^, ^56^ Research by Cortese et al. demonstrated the ineffectiveness of EEG neurofeedback for ADHD based on well‐controlled trials.^57^ However, studies by van der Kolk et al.^13^ and Young et al.^14^ reported significant reductions in PTSD symptoms and depressive symptoms, respectively, following EEG neurofeedback interventions.

#### fMRI neurofeedback

fMRI neurofeedback harnesses real‐time neuroimaging data to provide feedback on brain activity levels, facilitating self‐regulation. Notable findings from the reviewed papers include the efficacy of fMRI neurofeedback in chronic PTSD symptom improvement^13^ and increased amygdala activity in major depressive disorder (MDD).^58^ Additionally, studies by Koush et al.^59^ and Dehghani et al.^60^ explored connectivity‐based fMRI neurofeedback, demonstrating the modulation of emotion regulation networks and global brain connectivity during emotion regulation tasks.

#### fNIRS neurofeedback

fNIRS neurofeedback measures changes in cerebral blood flow and oxygenation using near‐infrared light, offering portability and accessibility compared to fMRI. While less prevalent in the reviewed papers, fNIRS neurofeedback holds promise in diverse applications, including ADHD, stroke rehabilitation, and cognitive enhancement. Research by Hohenfeld et al.^61^ showcased improved visuospatial memory in healthy elderly and prodromal Alzheimer's disease following fNIRS neurofeedback training.

#### Heart rate variability biofeedback

Heart rate variability (HRV) biofeedback focuses on modulating HRV to enhance stress resilience and emotional regulation. Although fewer studies in the reviewed papers explored HRV biofeedback, its potential in managing anxiety, hypertension, and stress‐related disorders was evident Schabus et al.^56^ conducted a double‐blind placebo‐controlled study on primary insomnia, revealing comparable efficacy between HRV biofeedback and placebo, highlighting the importance of nonspecific factors in treatment outcomes.

#### Neurofeedback gaming and VR

Integrating neurofeedback with gaming interfaces and VR environments enhances engagement and motivation during training sessions. While not extensively covered in the reviewed papers, neurofeedback gaming holds promise in neurorehabilitation and cognitive training. Scharnowski et al.^62^ demonstrated perceptual sensitivity enhancements through neurofeedback gaming, emphasizing the potential of interactive approaches in promoting self‐regulation.

#### Combined modalities and hybrid approaches

Some studies explored hybrid neurofeedback protocols combining multiple modalities, such as EEG–fMRI or EEG–fNIRS, to capitalize on their complementary strengths. These hybrid approaches offer enhanced spatial and temporal resolution, allowing for precise targeting of brain networks. While not as prevalent in the reviewed papers, studies by Haugg et al.^63^ and Alkoby et al. and Haugg et al.^49^, ^64^ investigated predictors of neurofeedback performance and identified factors influencing learning success across diverse study cohorts.

In addition to these modalities, researchers employed various experimental designs and methodologies, including randomized controlled trials and single‐case experimental designs, to rigorously investigate neurofeedback interventions. Standardized protocols and reporting guidelines, such as the consensus on the reporting and experimental design of clinical and cognitive‐behavioural neurofeedback studies (CRED‐nf checklist), contribute to methodological rigor and reproducibility across neurofeedback studies. Through continued innovation and interdisciplinary collaboration, researchers strive to unlock the full potential of neurofeedback in addressing clinical and cognitive‐behavioral challenges.

### Applications of neurofeedback

Neurofeedback, a versatile tool for modulating brain activity, finds application across diverse domains, ranging from clinical therapy to cognitive enhancement and sports performance.^53^, ^61^, ^65^, ^66^ Drawing insights from the summary and key findings of the reviewed papers encompassing a wide array of neurofeedback applications, we explore the various domains where neurofeedback has demonstrated efficacy and potential.

#### Clinical settings

Neurofeedback holds promise as a non‐invasive intervention for managing various neurological and psychiatric disorders.^40^, ^42^, ^47^, ^50^ Several studies in the reviewed papers investigated the efficacy of neurofeedback in clinical populations, including:

− ADHD: Despite initial optimism, well‐controlled trials, such as those by Cortese et al.,^57^ questioned the effectiveness of neurofeedback for ADHD symptom improvement. However, other studies, such as, van der Kolk et al.^13^ reported significant reductions in symptoms using neurofeedback interventions.
− PTSD: Research by van der Kolk et al.^13^ demonstrated the efficacy of neurofeedback in reducing chronic PTSD symptoms, offering a promising therapeutic approach for individuals with trauma‐related disorders.
− Depression and anxiety disorders: Studies by Young et al.^14^ and Schabus et al.^56^ explored the use of neurofeedback for depression and primary insomnia, respectively, highlighting its potential as a complementary or alternative treatment modality.

#### Cognitive enhancement

Beyond clinical populations, neurofeedback has garnered interest for its cognitive enhancement potential in healthy individuals and those seeking to optimize cognitive performance.^39^, ^48^ Papers reviewed in this domain explored:

− Memory Improvement: Hohenfeld et al.^61^ investigated the effects of neurofeedback on visuospatial memory in healthy elderly and prodromal Alzheimer's disease, suggesting a potential avenue for memory enhancement.
− Attention and Executive Functioning: While the efficacy of neurofeedback for ADHD remains debated, studies like the one by Cortese et al.^57^ contribute to our understanding of its role in attention regulation and executive functioning.

#### Sports training and performance

Neurofeedback has also garnered attention in sports psychology and athletic training,^46^, ^65^ offering a novel approach to enhancing performance and skill acquisition. While fewer studies in the reviewed papers focused on this application, the potential benefits of neurofeedback in sports training were evident:

− Performance optimization: By targeting specific neural networks implicated in motor control and performance, neurofeedback interventions have the potential to enhance athletes' cognitive and motor skills, contributing to improved sports performance.^45^, ^46^, ^65^
− Stress management: HRV biofeedback, a subset of neurofeedback, has been explored for stress resilience and emotional regulation in athletes, aiding in pre‐competition anxiety management and post‐game recovery.^58^

#### Personalized medicine and individualized therapy

With advancements in neuroimaging and machine learning, personalized neurofeedback protocols tailored to individuals' neural profiles are emerging.^6^, ^37^, ^63^ These personalized approaches offer targeted interventions and may yield better treatment outcomes by accounting for individual differences in brain functioning and responsiveness to neurofeedback.

#### Education and learning enhancement

While less explored in the reviewed papers, neurofeedback holds potential in educational settings for improving attention, concentration, and learning outcomes in students with attentional difficulties or learning disabilities. Further research in this domain could elucidate the role of neurofeedback in educational interventions and pedagogical practices.

In summary, neurofeedback demonstrates versatility in its applications, spanning clinical therapy, cognitive enhancement, sports training, and personalized medicine. While challenges remain, continued research and innovation in neurofeedback hold promise for addressing diverse neurological and cognitive‐behavioral challenges across populations and contexts.

### Advancements and innovations in neurofeedback

Neurofeedback, as a field, has witnessed significant advancements and innovations in recent years, driven by technological developments, methodological refinements, and interdisciplinary collaborations. Drawing insights from the summary and key findings of 65 papers, we explore the notable advancements and innovative approaches shaping the landscape of neurofeedback research and applications.

#### Technological developments

− Real‐time functional magnetic resonance imaging (rtfMRI): Papers such as Young et al.^14^ and Haugg et al.^63^ highlighted the use of rtfMRI neurofeedback for regulating brain activity in regions associated with mood disorders and cognitive functions. Advancements in rtfMRI techniques enable real‐time monitoring and modulation of neural activity, offering insights into brain–behavior relationships.
− EEG‐based neurofeedback systems: Innovations in EEG technology, including high‐density electrode arrays, wireless systems, and advanced signal processing algorithms, have facilitated the development of portable and user‐friendly EEG neurofeedback systems. Studies by Ordikhani‐Seyedlar et al.^66^ and Alkoby et al.^54^ underscored the potential of EEG‐based neurofeedback for enhancing attention, memory, and cognitive performance.

#### Machine learning and data analytics

− Predictive analytics: Advances in machine learning algorithms have enabled the identification of biomarkers and predictors of neurofeedback learning success. Haugg et al.^63^ demonstrated the use of machine learning mega‐analysis to predict neurofeedback performance based on pre‐training brain activity, highlighting the potential for personalized treatment approaches.
− Data‐driven analyses: Studies by Dehghani et al.^60^ and Haugg et al.^63^ employed data‐driven approaches to analyze brain connectivity patterns during emotion regulation and neurofeedback training. These analyses offer insights into the complex dynamics of brain networks and their modulation through neurofeedback interventions.

#### Integration with VR and gaming platforms

− VR‐based neurofeedback: Innovative studies, such as, Ordikhani‐Seyedlar et al.^66^ explored the integration of neurofeedback with VR environments to enhance user engagement and immersion during training sessions. VR‐based neurofeedback platforms offer interactive and customizable training scenarios, facilitating skill acquisition and behavior modification.
− Gamification of neurofeedback: By gamifying neurofeedback tasks and exercises, researchers have enhanced user motivation, compliance, and enjoyment during training sessions. Gamified neurofeedback systems leverage principles of reward‐based learning and reinforcement to promote skill acquisition and neuroplasticity.

#### Connectivity‐based neurofeedback

− Dynamic causal modeling (DCM): Papers by Koush et al.^59^ and Dehghani et al.^60^ introduced connectivity‐based neurofeedback approaches, leveraging techniques such as DCM to modulate interactions within distributed brain networks. Connectivity‐based neurofeedback allows for the targeted regulation of functional connectivity patterns, offering potential therapeutic benefits for psychiatric and neurological disorders.

#### Closed‐loop systems and adaptive protocols

− Adaptive neurofeedback protocols: Advances in closed‐loop neurofeedback systems enable real‐time adjustments to training parameters based on individual response patterns. These adaptive protocols optimize training efficacy and promote neuroplasticity by dynamically adjusting feedback signals in response to changes in brain activity.

#### Multimodal approaches

− Combination therapies: Integrating neurofeedback with other therapeutic modalities, such as cognitive‐behavioral therapy (CBT), mindfulness‐based interventions, and pharmacotherapy, enhances treatment outcomes and synergistically targets multiple dimensions of brain function and behavior.

In summary, advancements in technology, data analytics, and innovative methodologies have propelled the field of neurofeedback forward, expanding its applications and efficacy across diverse domains. Continued interdisciplinary collaboration and methodological innovation hold promise for further enhancing the effectiveness and accessibility of neurofeedback interventions.

### Key findings and efficacy of neurofeedback

The synthesis of findings from 65 papers provides valuable insights into the efficacy and outcomes of neurofeedback interventions across various clinical, cognitive, and behavioral domains. Here, we present the key findings and efficacy of neurofeedback based on the collective evidence from the reviewed literature.

#### Clinical applications

− ADHD: Cortese et al.^57^ reported that neurofeedback did not demonstrate effectiveness for ADHD based on well‐controlled trials. However, further exploration is warranted to refine protocols and assess learning outcomes comprehensively.
− PTSD: A randomized controlled trial by van der Kolk et al.^13^ found that neurofeedback significantly reduced PTSD symptoms compared to waitlist conditions, indicating its potential as an adjunctive therapy for chronic PTSD.

#### Cognitive enhancement

− Visual attention: Ordikhani‐Seyedlar et al.^66^ reviewed attention‐based brain–computer interfaces (BCIs) using EEG for neurofeedback therapy, highlighting the promising role of BCIs in treating attention disorders. Challenges remain in extracting attention‐related neural signals for optimal BCI performance.
− Emotion regulation: Koush et al.^59^ demonstrated that participants could learn to enhance emotion regulation capabilities through connectivity‐based neurofeedback, suggesting the therapeutic potential of this approach for mood disorders and emotional dysregulation (Table 2).

**Table 2:** Comparison of neurofeedback modalities: Resolution, evidence, challenges, and future potential.

Modality	Temporal resolution	Spatial resolution	Clinical evidence	Key challenges	Future potential
EEG	High (ms)	Low (cm‐level)	Strong evidence for ADHD, epilepsy, anxiety, and preliminary results in depression	Low spatial specificity; artifact sensitivity; standardization issues	Widely accessible; potential for wearable neurofeedback systems
fMRI	Low (s)	High (mm‐level)	Strong evidence in affective disorders, addiction, PTSD; growing in depression and schizophrenia	Expensive; limited accessibility; delay in BOLD response	High potential for individualized biomarkers and network‐based feedback
fNIRS	Moderate (s)	Moderate (cm‐level, mostly cortical)	Emerging evidence in ADHD, depression, and anxiety	Limited depth penetration; signal contamination from scalp	Portable and less expensive alternative to fMRI; promising for real‐world applications
MEG	High (ms)	High (mm‐level)	Limited but growing evidence	High cost; complex setup; less portable	Useful for precise network dynamics and source localization
Hybrid (EEG–fMRI, EEG–fNIRS)	Variable (depends on integration)	Combines strengths of modalities	Early but promising results in emotion regulation, cognitive training	Technical complexity; synchronization and signal integration issues	Potential to optimize both resolution and clinical outcomes

Abbreviations: ADHD, attention‐deficit/hyperactivity disorder; EEG, electroencephalography; fMRI, functional magnetic resonance imaging; fNIRS, functional near‐infrared spectroscopy; PTSD, post‐traumatic stress disorder.

#### Neurofeedback modalities

− fMRI neurofeedback: Young et al.^14^ conducted a randomized clinical trial on real‐time fMRI amygdala neurofeedback for MDD and found improvements in depressive symptoms and autobiographical memory recall. However, methodological challenges such as participant discomfort and motion artifacts were noted.
− EEG neurofeedback: Schabus et al.^56^ compared EEG neurofeedback with placebo for primary insomnia and found both to be equally effective, highlighting the importance of addressing nonspecific factors in treatment outcomes.

#### Predictors of success

− Machine learning analysis: Haugg et al.^63^ conducted a mega‐analysis to identify determinants of real‐time fMRI neurofeedback performance and improvement. Factors such as pre‐training runs and training patients over healthy participants were associated with better neurofeedback outcomes.

#### Challenges and limitations

− Methodological rigor: Fovet et al.^51^ emphasized the need for rigorous experimental design and caution in interpreting null results in neurofeedback research. Doubt exists on whether double‐blind designs alone can account for the variability in neurofeedback outcomes.

#### Future directions

− Personalized medicine: Alkoby et al.^54^ proposed personalized protocols based on resting‐state EEG data to improve cognitive functions in dyslexic children, suggesting a shift towards individualized neurofeedback interventions.
− Technological integration: Advances in VR, gaming platforms, and closed‐loop systems offer new avenues for enhancing user engagement and treatment adherence in neurofeedback therapy.

In conclusion, while neurofeedback shows promise as a therapeutic intervention for various clinical and cognitive conditions, further research is needed to address methodological challenges, optimize treatment protocols, and identify predictors of treatment response. The collective findings underscore the importance of interdisciplinary collaboration, technological innovation, and personalized approaches in advancing the field of neurofeedback therapy.

## LIMITATIONS AND CHALLENGES

Despite the promising findings and potential applications of neurofeedback, several limitations and challenges persist within the field.

### Heterogeneity in study designs

Many of the reviewed studies exhibit heterogeneity in study designs, including variations in sample sizes, control conditions, and outcome measures. For instance, studies such as “Neurofeedback for attention‐deficit/hyperactivity disorder: Meta‐analysis of clinical and neuropsychological outcomes” by Cortese et al.^57^ and “A randomized controlled study of neurofeedback for chronic PTSD” by van der Kolk et al.^13^ employ different methodologies and outcome measures, making direct comparisons challenging.

### Small sample sizes

A significant number of studies included in this review have relatively small sample sizes, limiting the generalizability of their findings. Studies such as “Randomized clinical trial of real‐time fMRI amygdala neurofeedback for major depressive disorder: Effects on symptoms and autobiographical memory recall” by Young et al.^14^ and “Improvement of neurofeedback therapy for improved attention through facilitation of brain activity using local sinusoidal extremely low frequency magnetic field exposure” by Mehran et al.^53^ often involve small cohorts, which may not adequately represent the broader population.

### Lack of long‐term follow‐up

Many studies have short‐term follow‐up periods, hindering the assessment of the long‐term efficacy and sustainability of neurofeedback interventions. For example, “A randomized controlled study of neurofeedback for chronic PTSD” by van der Kolk et al.^13^ reports only a 1‐month follow‐up period, limiting conclusions about the permanency of neurofeedback effects.

### Variability in neurofeedback protocols

The lack of standardization in neurofeedback protocols across studies poses a challenge to comparing results and establishing consistent best practices. While some studies, like “Learning control over emotion networks through connectivity‐based neurofeedback” by Koush et al.^59^ explore innovative protocols such as connectivity‐based neurofeedback, the diversity of approaches complicates efforts to identify optimal intervention strategies.

### Placebo and expectation effects

Addressing placebo and expectation effects remains a significant challenge in neurofeedback research. Studies like “Better than sham? A double‐blind placebo‐controlled neurofeedback study in primary insomnia” by Schabus et al.^56^ highlights the difficulty in distinguishing between specific treatment effects and nonspecific placebo responses, underscoring the need for rigorous control conditions and blinding procedures.

### Interpretation of neural mechanisms

While neurofeedback studies demonstrate behavioral improvements, elucidating the underlying neural mechanisms remains a challenge. Studies such as “On assessing neurofeedback effects: Should double‐blind replace neurophysiological mechanisms?” by Fovet et al.^51^ emphasize the importance of understanding the neural substrates of neurofeedback effects to optimize intervention protocols and target specific brain networks effectively.

### Ethical considerations

The ethical implications of neurofeedback interventions, particularly concerning vulnerable populations such as children and individuals with psychiatric disorders, warrant careful consideration. Studies like “Can we predict who will respond to neurofeedback? A review of the inefficacy problem and existing predictors for successful EEG neurofeedback learning” by Alkoby et al.^54^ underscore the importance of personalized approaches and minimizing potential harms associated with neurofeedback interventions.

Addressing these limitations and challenges will be crucial for advancing the field of neurofeedback and maximizing its potential benefits for clinical practice and cognitive enhancement.

## FUTURE DIRECTIONS AND POTENTIAL OF NEUROFEEDBACK

The exploration of neurofeedback has uncovered promising avenues for future research and applications, as evidenced by the findings synthesized from the corpus of 65 papers. These insights pave the way for advancements in both clinical practice and scientific inquiry, offering opportunities to enhance therapeutic interventions and deepen our understanding of brain function.

One notable direction for future research involves the refinement of neurofeedback protocols and methodologies to optimize treatment outcomes across diverse populations. Studies such as “Neurofeedback for attention‐deficit/hyperactivity disorder: Meta‐analysis of clinical and neuropsychological outcomes from randomized controlled trials”^57^ underscore the importance of standardizing protocols and assessing learning mechanisms to improve the effectiveness of neurofeedback interventions. By incorporating personalized approaches and tailoring protocols to individual patient characteristics, researchers can enhance treatment efficacy and address the heterogeneity of treatment responses observed in clinical trials.

Moreover, advancements in technology and data analytics hold promise for expanding the scope and applicability of neurofeedback interventions. The integration of machine learning algorithms, as demonstrated in “Determinants of real‐time fMRI neurofeedback performance and improvement—A machine learning mega‐analysis,”^63^ enables the identification of factors influencing neurofeedback success and the development of predictive models for treatment outcomes. By harnessing the power of big data and computational modeling, researchers can uncover novel insights into brain dynamics and develop personalized neurofeedback strategies tailored to individual patient needs.

Additionally, the future of neurofeedback research lies in its integration with other therapeutic modalities and interdisciplinary approaches. Studies such as “Learning control over emotion networks through connectivity‐based neurofeedback”^52^, ^59^, ^67^ highlight the potential of combining neurofeedback with cognitive‐behavioral techniques and pharmacological interventions to enhance treatment efficacy and promote long‐term neuroplasticity. By leveraging synergies between different treatment modalities, researchers can develop comprehensive intervention protocols that target multiple dimensions of brain function and behavior, leading to more holistic and personalized treatment approaches.

Furthermore, the adoption of neurofeedback in emerging fields such as VR and augmented reality (AR) opens up new possibilities for immersive and interactive therapeutic interventions. Research such as “Improvement of neurofeedback therapy for improved attention through facilitation of brain activity using local sinusoidal extremely low‐frequency magnetic field exposure”^53^ suggests that combining neurofeedback with VR/AR technologies can enhance engagement, motivation, and treatment outcomes, particularly in pediatric populations and individuals with attention‐related disorders. By harnessing the immersive nature of VR/AR environments, researchers can create dynamic and interactive neurofeedback experiences that promote learning, engagement, and neuroplasticity, thereby enhancing treatment outcomes and patient satisfaction.

In conclusion, the future of neurofeedback holds great promise for revolutionizing clinical practice, advancing scientific understanding, and improving patient outcomes. By embracing personalized approaches, leveraging technological innovations, and fostering interdisciplinary collaborations, researchers can unlock the full potential of neurofeedback as a powerful tool for modulating brain function, enhancing cognitive performance, and promoting mental health and well‐being.

## CONCLUSION

In this comprehensive review, we have examined the applications, advancements, and future directions of neurofeedback, drawing insights from a synthesis of 65 seminal papers in the field. Our exploration has illuminated the multifaceted landscape of neurofeedback research, highlighting its versatility as a therapeutic intervention and its potential to transform our understanding of brain function and cognition.

Through an analysis of key findings and limitations across diverse domains, including clinical psychology, cognitive neuroscience, and sports performance, we have gained valuable insights into the efficacy and challenges of neurofeedback interventions. Studies such as “A randomized controlled study of neurofeedback for chronic PTSD”^13^ and “Randomized clinical trial of real‐time fMRI amygdala neurofeedback for major depressive disorder”^14^ underscore the efficacy of neurofeedback in alleviating symptoms of psychological disorders and improving emotional regulation capacities. However, challenges such as the lack of standardization in protocols and the variability in treatment responses emphasize the need for continued research and innovation in the field.

Furthermore, our review has shed light on recent advancements and innovations in neurofeedback technology, including the integration of machine learning algorithms, VR environments, and connectivity‐based neurofeedback techniques. Studies such as “Determinants of real‐time fMRI neurofeedback performance and improvement—A machine learning mega‐analysis”^63^ and “Learning control over emotion networks through connectivity‐based neurofeedback”^59^ highlight the potential of these novel approaches to enhance treatment efficacy and promote neuroplasticity.

Looking ahead, the future of neurofeedback holds great promise for revolutionizing clinical practice and advancing scientific understanding. By embracing personalized approaches, leveraging technological innovations, and fostering interdisciplinary collaborations, researchers can unlock the full potential of neurofeedback as a powerful tool for modulating brain function, enhancing cognitive performance, and promoting mental health and well‐being.

In conclusion, this review underscores the transformative impact of neurofeedback on clinical psychology, neuroscience, and human performance. By harnessing the power of neurofeedback, we have the opportunity to not only alleviate symptoms of psychological disorders but also unlock the latent potential of the human brain to thrive and flourish.

## AUTHOR CONTRIBUTIONS

**Hassan Jubair**: Conceptualization; formal analysis; validation; project administration; writing—original draft; writing—review and editing. **Mithela Mehenaz**: Data curation; software; resources; validation; writing—original draft; writing—review and editing. **Md. Merajul Islam**: Software; resources; validation; writing—review and editing. **Nilufa Yeasmin**: Resources; validation; methodology; writing—review and editing.

## CONFLICT OF INTEREST STATEMENT

The authors declare no conflicts of interest.

## ETHICS APPROVAL STATEMENT

N/A.

## PATIENT CONSENT STATEMENT

N/A.

## CLINICAL TRIAL REGISTRATION

N/A.

## Data Availability

Data sharing is not applicable to this article as no datasets were generated or analyzed during the current study.
